# Modeling and correct the GC bias of tumor and normal WGS data for SCNA based tumor subclonal population inferring

**DOI:** 10.1186/s12859-018-2099-0

**Published:** 2018-04-11

**Authors:** Yanshuo Chu, Mingxiang Teng, Yadong Wang

**Affiliations:** Center for Bioinformatics, Harbin Institute of Technology, Harbin, China

**Keywords:** Somatic copy number alternation, Subclonal frequency, GC bias

## Abstract

**Background:**

Somatic copy number alternations (SCNAs) can be utilized to infer tumor subclonal populations in whole genome seuqncing studies, where usually their read count ratios between tumor-normal paired samples serve as the inferring proxy. Existing SCNA based subclonal population inferring tools consider the GC bias of tumor and normal sample is of the same fature, and could be fully offset by read count ratio. However, we found that, the read count ratio on SCNA segments presents a Log linear biased pattern, which influence existing read count ratios based subclonal inferring tools performance. Currently no correction tools take into account the read ratio bias.

**Results:**

We present Pre-SCNAClonal, a tool that improving tumor subclonal population inferring by correcting GC-bias at SCNAs level. Pre-SCNAClonal first corrects GC bias using Markov chain Monte Carlo probability model, then accurately locates baseline DNA segments (not containing any SCNAs) with a hierarchy clustering model. We show Pre-SCNAClonal’s superiority to exsiting GC-bias correction methods at any level of subclonal population.

**Conclusions:**

Pre-SCNAClonal could be run independently as well as serving as pre-processing/gc-correction step in conjuntion with exsiting SCNA-based subclonal inferring tools.

**Electronic supplementary material:**

The online version of this article (10.1186/s12859-018-2099-0) contains supplementary material, which is available to authorized users.

## Background

Tumor heterogeneity introduces challenges in cancer tissue diagnosis and subsequent treatment [1]. Currently, projects such as TCGA [2] screened thousands of tumor samples using whole-genome sequencing(WGS) on tissue (bulk) cells, provide more explicit molecular insights on identifying cancer cell types and sub-types than other bioinformatics methods [3–6]. To decipher cell composition in bulk cell WGS, somatic copy number alterations (SCNAs), commonly found in tumor cells [7], are utilized as the representative to determine tumor subclonal populations in a tumor-normal tissue paired manner by existing tools, e.g. MixClone [8], THetA [9]. The benefits of using SCNAs is that the WGS data doesn’t have to be deeply sequenced [8]. However, existing tools lack the ability to properly account for sequencing GC bias which is widely observed in DNA-seq data [10].

Evidences have showed that GC-bias could affect SCNA identification in tumor cells [11–13]. Existing tools consider the SCNA segments have the same sequence properties between the normal and tumor samples, and consider the bias could be offset to use the read count ratios between tumor and normal paired samples [8, 9]. However, We found that, in a GC biased study, the GC contents and read count ratios on SCNA segments present a Log linear biased pattern. Though existing method [14] suggests removing GC bias by modeling GC cotent with tumor-normal coverage difference for small genomic windows, however, we find that small window is not a proper and robust resolution for SCNA.

We present Pre-SCNAClonal, a tool that improving tumor subclonal population inferring by correcting GC-bias at SCNAs level. Pre-SCNAClonal first corrects GC bias using Markov chain Monte Carlo probability model, then accurately locates baseline DNA segments (not containing any SCNAs) with a hierarchy clustering model. We show Pre-SCNAClonal’s superiority to exsiting GC-bias correction methods for SCNA-based tumor reconstruction tools at any level of subclonal population. We also note that Pre-SCNAClonal could be run independently as well as serving as pre-processing/gc-correction step in conjuntion with exsiting SCNA-based subclonal inferring tools.

## Data

The WGS data of human breast cancer HCC2218 and HCC1954 with different levels of normal contamination (coverage 30x) are used to validate the method proposed in this paper. Each of the HCC1954 samples, HCC1954.mix1.n5t95, HCC1954.mix1.n20t80, HCC1954.mix1.n40t60, HCC1954.mix1.n60t40, HCC1954.mix1.n80t20 and HCC1954.mix1.n95t5, contains one tumor subclone. The tumor subclonal frequencies (or tumor purity) of these samples are 0.95, 0.80, 0.60, 0.40, 0.20 and 0.05, respectively. We also use the data of human ovary cancer sample TCGA-13-0723 in Benjamini’s work [11] to show the read count ratio’s GC bias between paired tumor and normal sample.

The WGS sequence alignment data (.bam files) of HCC2218 and its paired normal sample are publicly available on Illumina BaseSpace Sequence Hub website https://basespace.illumina.com. The WGS sequence alignment data (.bam files) of HCC1954 and its paired normal sample and the WGS data with different levels of normal contamination are public available at National Cancer Institute GDC Data Portal https://gdc.cancer.gov/resources-tcga-users/tcga-mutation-calling-benchmark-4-files. The WGS sequence alignment data (.bam files) of TCGA-13-0723 is available at National Cancer Institute GDC Data Portal https://portal.gdc.cancer.gov/ only for authorized user.

## Methods

### GC bias of the tumor WGS data does not have the same feature as its paired normal

Let coefficient *θ*_*j*_ denote the effect of mappability and genomic length of segment *j*, $\bar {C}_{j}$ denote the average copy number of segment *j*, *λ*_*j*_ denote the expected read counts, and let $D_{j}^{N}$ denote the read counts of segment *j* in matched normal genome, then for segment *i* and segment *j*, existing SCNA based tumor subclonal populations inferring tools [8, 9] assume that ${\lambda _{i}}/{\lambda _{j}} ={\bar {C}_{i}\theta _{i}}/{\bar {C}_{j}\theta _{j}}$, and $\theta _{i} / \theta _{j} = D_{i}^{N} / D_{j}^{N}$, then

1 $$ \frac{D_{i}^{S}}{D_{j}^{S}} = \frac{\lambda_{i}}{\lambda_{j}} = \frac{\bar{C}_{i}\theta_{i}}{\bar{C}_{j}\theta_{j}} = \frac{\bar{C}_{i}}{\bar{C}_{j}} * \frac{D_{i}^{N}}{D_{j}^{N}}.  $$

**Fig. 1:**
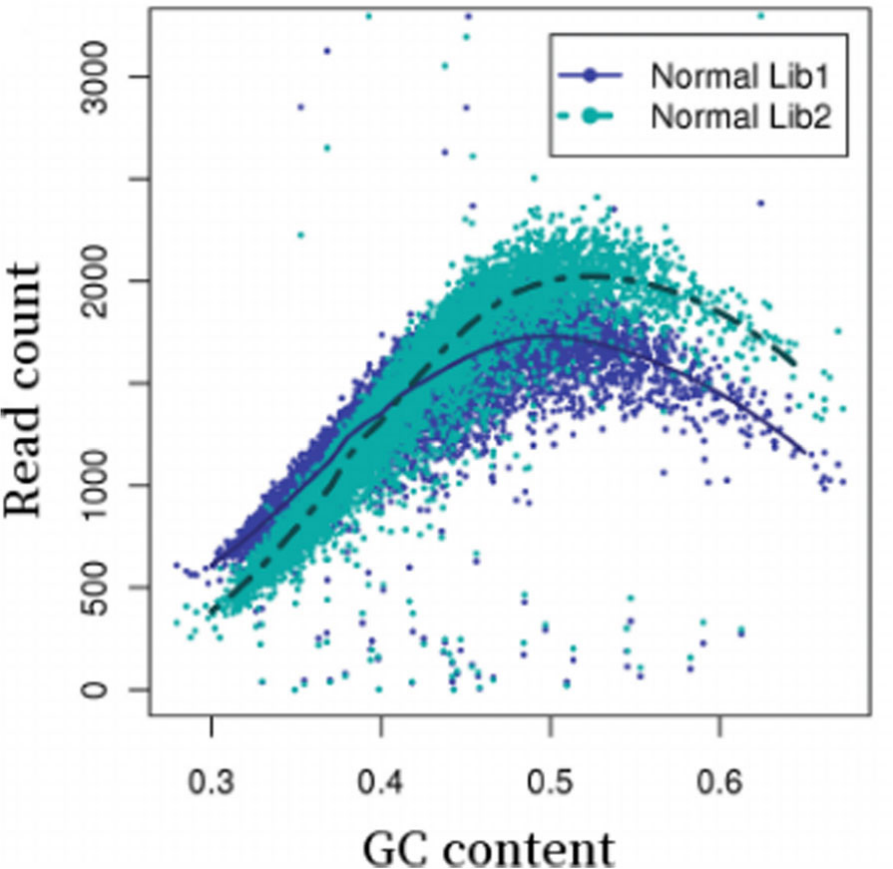
GC curves (10 kb bins). Observed fragment counts and loess lines plotted against GC of two libraries from the same normal sample TCGA-13-0723.Bins were randomly sampled from chromosome 1. This Figure is drawn by Benjamini et al. [11]

Figure 1 shows the two normal libraries from the same normal sample, and there is a crossover point of the two loess lines. Here we suppose the normal Lib 2 is a tumor sample has no variations, and normal Lib 1 is its paired normal sample. According to Eq. 1,

2 $$ \frac{D_{i}^{Lib2}}{D_{j}^{Lib2}} = \frac{\lambda_{i}}{\lambda_{j}} = \frac{\bar{C}_{i}\theta_{i}}{\bar{C}_{j}\theta_{j}} = \frac{2}{2} * \frac{D_{i}^{Lib1}}{D_{j}^{Lib1}} = \frac{D_{i}^{Lib1}}{D_{j}^{Lib1}},  $$

If *j* is the crossover point, we have $D_{i}^{Lib2} = D_{i}^{Lib1}$, which means the two loess lines should overlap each other. This demonstrates that the GC bias is different in the tumor and its paired normal sample.

### Modelling the difference of GC bias between paired tumor and normal sample

We find that, the difference between the GC bias of tumor and its paired normal could be modelled as following equation,

3 $$ \begin{aligned} &D_{i}^{N} = \frac{f(GC_{i})}{\exp(a_{1} * GC_{i}) / (d_{1} * GC_{i})} \\ &D_{i}^{S} = \frac{f(GC_{i})}{\exp(a_{2} * GC_{i}) / (d_{2} * GC_{i})} \\ \end{aligned},  $$

**Fig. 2:**
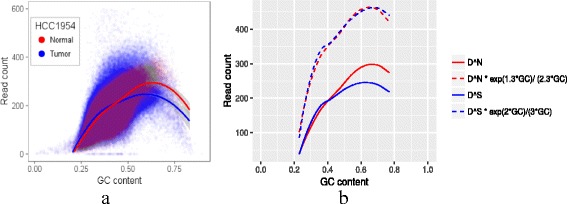
The relationship of GC bias between paired tumor and normal samples. **a** The GC bias distribution of read count observed in 500bp bins with high mappability (top 10%). To account for uniqueness of sequences, a mappability measure is calculated for each position (base pair) in the bin. A location is called ‘mappable’ if the k-mer of the reference genome starting at the location is not perfectly repeated at any other location in the genome, where k is the read length. Both of the tumor and normal samples are processed by Illumina platform to produce the reads, and use GATK’s table recalibration and use Burrows-Wheeler Aligner (bwa) to align the sequence data with the same parameters. **b** In this figure, the red and blue solid lines are the mean functions of loess smooth in (**a**). The red and blue dashed lines are the mean functions of loess smooth in (**a**) multiplies exp(1.3∗*GC*)/(2.3∗*GC*) and exp(2∗*GC*)/(3∗*GC*) respectively

In this equation, *f*(*GC*_*i*_) is a function of GC content, which represents the bias feature that shared by tumor and its paired normal sample. *a*_1_, *a*_2_, *d*_1_ and *d*_2_ denote the distinctions of bias feature between tumor and its paired normal sample. *a*_1_ and *a*_2_ represent the curvature of tumor and its paired normal sample respectively; *d*_1_ and *d*_2_ represent the distance of tumor and its paired normal sample respectively; As shown in Fig. 2, the distinctions of bias feature between the paired tumor sample HCC1954 and its paired normal HCC1954 BL could be well captured by this model.

According to Eq. 3, Eq. 1 is transformed into

4 $$ \frac{D_{i}^{S}}{D_{j}^{S}} = \frac{\bar{C}_{i}}{\bar{C}_{j}} * \exp\left[(a_{2}-a_{1})* (GC_{j} - GC_{i})\right] * \frac{D_{i}^{N}}{D_{j}^{N}},  $$

then,

5 $$ \log{\frac{D_{i}^{S}}{D_{i}^{N}}} - \log{\frac{D_{j}^{S}}{D_{j}^{N}}} = \log{\frac{\bar{C}_{i}}{\bar{C}_{j}}} + \left(a_{2}-a_{1}\right)* \left(GC_{j} - GC_{i}\right).  $$

Equation 5 reveals that the read count ratio presents a Log linear biased pattern on SCNAs which we will prove it later. Equation 5 also shows that the read count ratio’s GC bias between paired tumor and normal sample exists if the curvature of tumor and its paired normal sample are not the same. We also find this phenomenon in HCC2218 (Additional file 1: Figure S1).

### BAF in tumor WGS data presents symmetrical pattern in [0,1] at heterozygous SNP sites

Let *μ*_*i*_ denote the BAF of SCNA segment *i* of tumor genome on germline heterozygous SNP site, and let *C*_*i*_, *G*_*i*_ respectively denote the absolute copy number and genotype of SCNA segment *i*. The B allele (non-reference allele) could be either maternal or paternal allele, thus the BAF of SCNA segments of tumor genome presents symmetrical pattern in [ 0,1] (please see Additional file 1: Supplementary 3.3.2 for detail proof). Let *ξ*_*i*_ denote the BAF of the tumor sample, *ϕ*_*i*_ denote the subclonal population frequency, then,

6 $$ \xi_{i} = \frac{\phi_{i} * C_{i} * \mu_{i} + \left(1- \phi_{i}\right) *2*\frac{1}{2}}{\bar{C}_{i}},  $$

7 $$ \bar{C}_{i} = \phi_{i} * C_{i} + \left(1- \phi_{i}\right) *2.  $$

In Eqs. 6 and 7, ‘2’ and ‘$\frac {1}{2}$’ are the copy number and heterozygous BAF of normal sample. Then, *ξ*_*i*_ is symmetrical in [ 0,1], because *μ*_*i*_ is symmetrical in [ 0,1].

### GC bias of read count ratio affects SCNA based subclonal population analysis

**Fig. 3:**
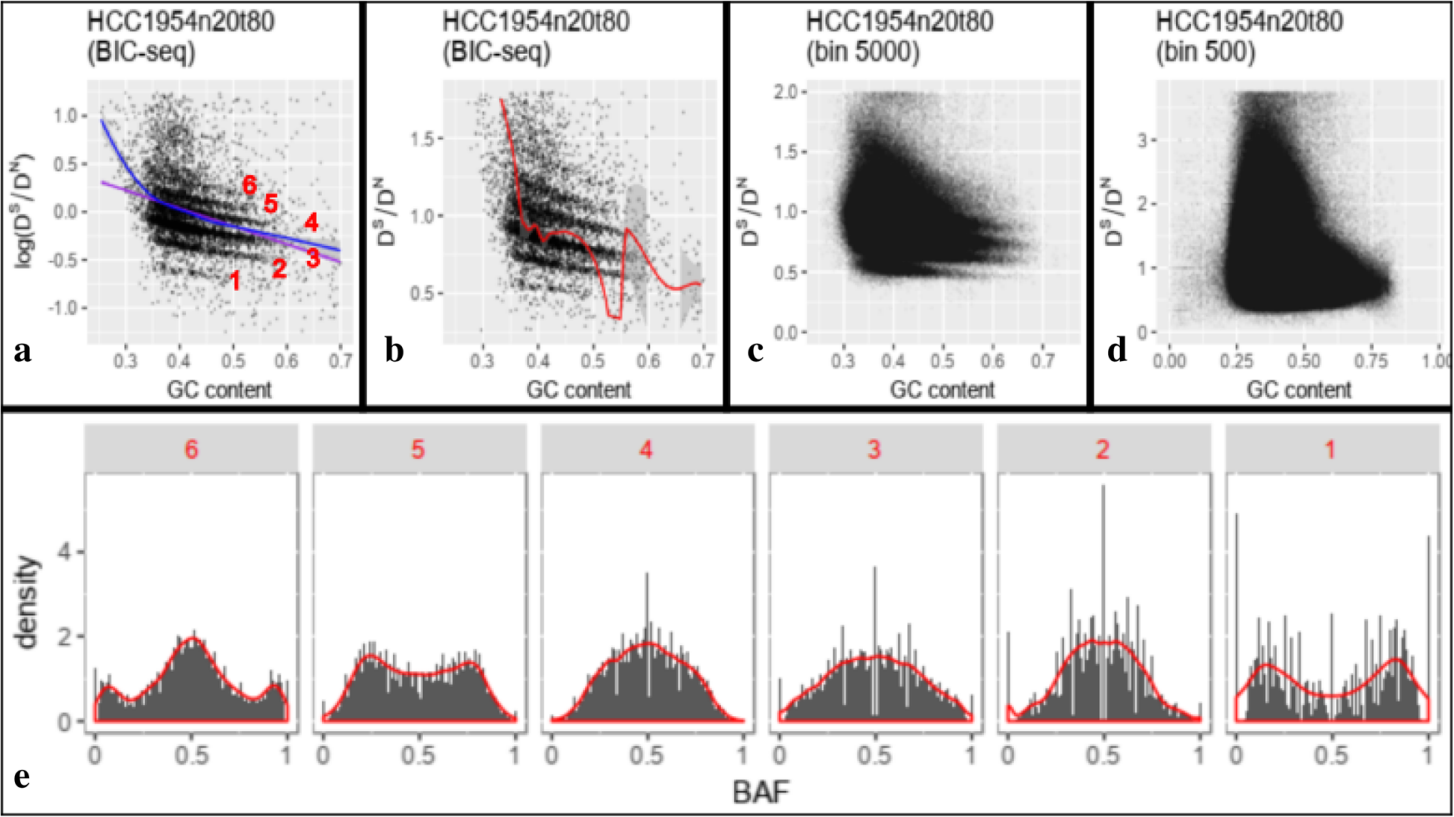
GC bias of WGS data of tumor-normal paired sample HCC1954.mix1.n20t80 of TCGA mutation calling benchmark 4. Let *D*^*S*^ and *D*^*N*^ respectively denote the read counts of the segment of tumor and normal samples. **a** The GC bias of the Log ratio of tumor and normal read counts of the SCNA segments. The purple and blue lines are linear regression and loess regression lines respectively. **b** The GC bias of the ratio of tumor and normal read counts of the SCNA segments. The red line are drawn by the loess regression model with a quadratic polynomial function, which is used to rectify the distribution of the ratio *D*^*S*^/*D*^*N*^ in the state-of-art GC correction method [14]. **c** The GC bias of the ratio of tumor and normal read counts of the 5000 bp bin. Since the majority (81%) of CNV calls are between 1 kb and 100 kb [17], most of 5000 bp bins spans only one SCNA. This sub-figure shows most SCNAs clustered clearly into multiple strips. **d** The GC bias of the ratio of tumor and normal read counts of the 500 bp bin. **e** The distribution of B-allele frequency (BAF) of stripe 1–6 in Fig. 3a. The SCNA segments are obtained by BIC-seq [18]

**Fig. 4:**
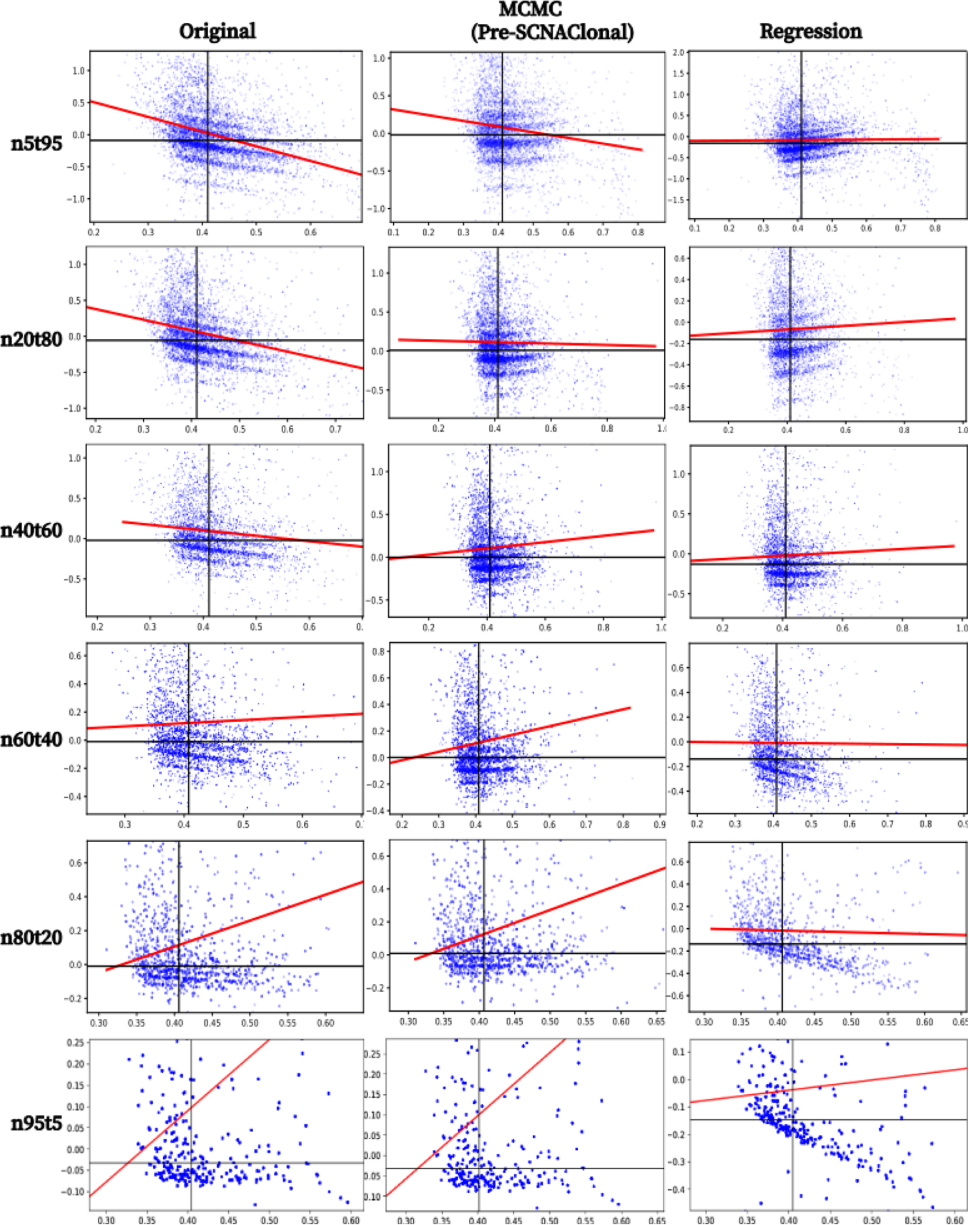
Read count ratio’s GC bias correction of HCC1954 with different levels of normal contamination. Here ‘n5t95’, ‘n20t80’, ‘n40t60’, ‘n60t40’ and ‘n95t5’ respectively denote the tumor sample ‘HCC1954.mix1.n5t95’, ‘HCC1954.mix1.n20t80’, ‘HCC1954.mix1.n40t60’, ‘HCC1954.mix1.n60t40’, ‘HCC1954.mix1.n80t20’ and ‘HCC1954.mix1.n95t5’. Subfigures in the ‘Origin’ column show the GC bias of read count ratio before correction, and column ‘MCMC’ and ‘Regression’ show the GC bias of read count ratio after the correction by MCMC model of Pre-SCNAClonal and Regression model respectively. The red lines are the linear regression lines. All the subfigures are plotted by Pre-SCNAClonal

By increasing the window size to 5000bp (Fig. 3c) or even larger at SCNA level (Fig. 3b), the 2D plot between GC content and tumor-normal coverage ratio clearly clustered into multiple stripes. It is noted that the relationship is pretty linear between GC content and log ratio of tumor-normal coverage on SCNAs (Fig. 3a) and we show that slopes of linear relation vary across tumors (Additional file 1: Figure S1). We also show that the gaps between the stripes in Fig. 3a are proportional to the subclonal populations (as shown in the sub-figures in the first column of Fig. 4). The SCNA segments which are clustered into the same stripe, present the symmetrical pattern of B allele frequency (BAF) density on the heterozygous allele loci of paired normal sample (Fig. 3e), which reveals that these SCNA segments in the same stripe contain the same copy number(see Additional file 1: Supplementary 3.3.2 for detail proof). While using the ratio of read counts of SCNA segments to get the precise subclonal population of each SCNA, it needs to correct the GC bias of the gap first.

### Existing read count ratio’s GC bias correction methods are not suitable for SCNA based subclonal population analysis

Existing GC correction methods for WGS data of tumor normal paired sample, such as CNAnorm [14], rectifies the distribution of the ratio of read counts of the small window, aiming at finding the position of SCNA and absolute copy number (Fig. 3d) by merging the adjoining small window with similar ratio properties. This method uses regression model to rectify the GC content distribution of the ratio and hence removing the dependencies on GC content. However, while using this GC correction method to rectify the bias of read count ratio for SCNA based subclonal population analysis, it additionally requires the regression correctly capture the slope of the gaps between the SCNA stripes. As shown in Fig. 3a and b, linear or loess regression could be easily biased by outliers, regression lines in Fig. 3a and b do not parallel the stripes, hence there would still exist GC content bias after removing the dependencies on GC content based on these regression lines (see Fig. 4).

### Models of Pre-SCNAClonal for read count ratio’s GC bias correction for SCNA based subclonal population analysis

#### MCMC model

Pre-SCNAClonal uses a Markov chain Monte Carlo (MCMC) model to pick out the maximum posterior probability of stripe slope *m* listed in Eq. 8,

8 $$  \begin{array}{ll} p(m|Y,X) & \sim p(m) * p(Y,X|m)\\ m & \sim \text{Uniform}(a- \delta, a+\delta)\\ p(Y, X|m) & = \Lambda(D, \tau * \max(cn))\\ D & = density(Y')\\ Y' & = Y-(m*X + c) + \text{median}(Y)\\ \end{array},  $$

here *Y*, *X* denotes log(*D*^*S*^/*D*^*N*^) and GC content respectively; *a*, *c* are slope and intercept pre-determined by two points, coordinates of which are the median of *Y* and *X* at high and low GC content areas; *δ* is the slope range pre-specified; *D* denotes the density function, *Λ*(*D*,*τ*∗ max(*cn*)) denotes the sum of top (largest) *τ*∗ max(*cn*) peaks of density curve of *D*; *τ* denotes the number of subclonal populations, max(*cn*) denotes the maximum copy number pre-defined. *Y*^′^ represents the corrected *Y*.

#### Hierarchy clustering model

Note that, normally, the read counts of tumor segments without SCNA (defined as baseline) are not equivalent to those from paired normal samples due to coverage difference. According to Eqs. 6 and 7, the $\bar {C}_{i}$ and *ξ*_*i*_ of baseline segment always equals to 2 and $\frac {1}{2}$ respectively. If and only if $\mu _{i}=\frac {1}{2}$, $\xi _{i} = \frac {1}{2}$. Then according to Eqs. 5 and 7, the baseline segments locate in the SCNA stripe with $\xi _{i} = \frac {1}{2}$ and the smallest $\log {\frac {D_{i}^{S}}{D_{i}^{S}}}$, because only positive even *C*_*i*_ with equal paternal and maternal copy could make $\mu _{i}=\frac {1}{2}$. Thus, after the GC correction, Pre-SCNAClonal picks out all the segments with $\xi _{i} = \frac {1}{2}$, and imports a hierarchy clustering model to group the segments into several clusters, then Pre-SCNAClonal selects the cluster with smallest $\log {\frac {D_{i}^{S}}{D_{i}^{S}}}$ as baseline segments.

## Results

We use WGS data of HCC1954 with different levels of normal contamination (coverage 30x) to test the models of Pre-SCNAClonal.

### Result of MCMC model

As shown in Fig. 4, MCMC model of Pre-SCNAClonal could better correct the read count ratio’s GC bias than linear regression method [14]. The GC correction results of linear regression are either over-corrected (HCC1954.mix1.n5t95 and HCC1954.mix1.n20t80) or under-corrected(HCC1954.mix1.n60t40, HCC1954.mix1.n80t20 and HCC1954.mix1.n95t5).

To further test MCMC model, we also develop a package that could simulate data with extreme bias (please refer to Additional file 1: Supplementary 2.1 for detail). Results show that the MCMC model of Pre-SCNAClonal is robust and noise tolerant, outperforms the regression method in CNAnorm [14].

### Result of hierarchy clustering model

**Fig. 5:**
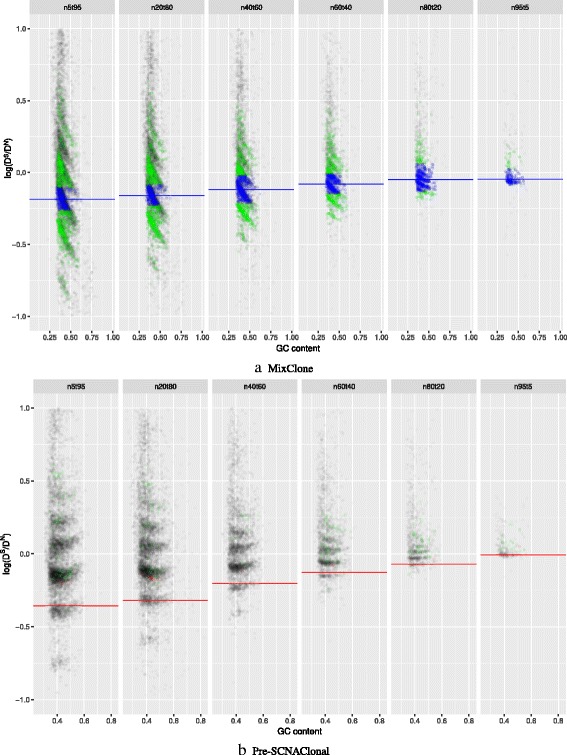
Distribution of log*D*^*S*^/*D*^*N*^ and baseline segments. **a** The green and blue points together are the segments without LOH, the blue points are the baseline segments selected by MixClone, The blue line in each sub-figure is the average value of ${D_{j}^{S}}/{D_{j}^{N}}$ of baseline segments. **b** The green and blue points together are the segments with no LOH and B allele frequencies around 0.5, the red points are the baseline segments selected by Pre-SCNAClonal. The red line in each sub-figure is the average value of log*D*^*S*^/*D*^*N*^ of baseline segments. All the points in this figure are plotted by ggplot2 R package with opacity parameter *α*=0.05

The baseline selection method in MixClone [8] obtains baseline by removing outliers of read count ratios of the segments that do not lose heterzygosity(LOH). In the WGS data, it is difficult to distinguish LOH from sequencing deviation or error. As shown in Fig. 5a, segments that do not lose heterzygosity are randomly distributed everywhere. Baseline selection method of MixClone almost picks out all the segments as baseline while the tumor purity is low. In comparison, as shown in Fig. 5b, baseline obtained by Pre-SCNAClonal is lower and more consistent than the baseline obtained by MixClone.

We calculate the ploidy number based on baseline segments’ $\log {\frac {D_{i}^{S}}{D_{i}^{N}}}$ to validate baseline selection model of Pre-SCNAClonal, and the result shows that the tumor sample HCC1954 is tetraploidy which is the same as results of COSMIC [15] and ABSOLUTE [16](for detail procedure, please see Additional file 1: Supplementary 3.3.1). Furthermore, the BAF distribution on germline heterozygous SNP site also shows the baseline segments obtained by hierarchy clustering models are correct (for detail procedure, please see Additional file 1: Supplementary 3.3.2).

### Result of pipeline test

We respectively string Pre-SCNAClonal with two typical SCNAs based subclonal inferring tools, MixClone [8] and THetA [9], to test the bias correction and baseline selection models of Pre-SCNAClonal on HCC1954. As shown in Table 1, Pre-SCNAClonal–MixClone pipeline almost precisely estimated the subclonal frequency for all HCC1954 tumor samples, which outperforms MixClone alone a lot. Pre-SCNAClonal–THetA pipeline also provided better estimation than THetA alone (THetA could not run on sample ‘n80t20’ and ‘n95t5’ for their BIC-seq segments number lower than 1000). This result shows that Pre-SCNAClonal could greatly improve the performance of tumor subclonal population inferring algorithms.

**Table 1:** Pipeline test of Pre-SCNAClonal on HCC1954

Sample name	n5t95	n20t80	n40t60	n60t40	n80t20	n95t5
Pre-SCNAClonal–MixClone (%)	0.874	0.722	0.523	0.374	0.200	0.054
MixClone (%)	0.645	0.589	0.471	0.199	0.144	0.188
Pre-SCNAClonal–THetA (%)	0.572	0.461	0.281	0.163	-	-
THetA (%)	0.463	0.374	0.269	0.148	-	-

Here ‘n5t95’, ‘n20t80’, ‘n40t60’, ‘n60t40’ and ‘n95t5’ respectively denote the tumor sample ‘HCC1954.mix1.n5t95’, ‘HCC1954.mix1.n20t80’, ‘HCC1954.mix1.n40t60’, ‘HCC1954.mix1.n60t40’, ‘HCC1954.mix1.n80t20’ and ‘HCC1954.mix1.n95t5’. Each of these sample contains one tumor subclone. Numbers in the table are the tumor subclonal frequencies predicted by the pipeline

## Discussion

**Fig. 6:**
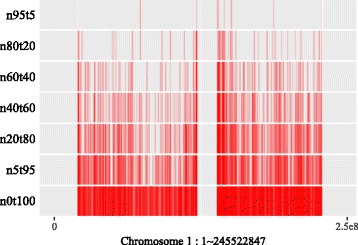
Breakpoints distribution on chromosome 1 of mixed “HCC1954” samples. Here ‘n5t95’, ‘n20t80’, ‘n40t60’, ‘n60t40’ and ‘n95t5’ respectively denote the tumor sample ‘HCC1954.mix1.n5t95’, ‘HCC1954.mix1.n20t80’, ‘HCC1954.mix1.n40t60’, ‘HCC1954.mix1.n60t40’, ‘HCC1954.mix1.n80t20’ and ‘HCC1954.mix1.n95t5’. ‘n0t100’ denotes the tumor sample ‘HCC1954’ contains no normal contamination. Each of these sample contains one tumor subclone. All the breakpoints are obtained by BIC-seq [18]

Generally, SCNAs with larger subclonal frequency could be more precisely located relatively. However, due to the twice sequencing procedures of tumor and its paired normal, the read information of the genomic regions with the same copy number in tumor sample is not exactly the same as its paired normal’s. Moreover, the lower read overage of next generation sequencing (NGS) makes the perturbation more likely to be mistaken for a SCNA. As shown in Fig. 6, the number of SCNA breakpoints obtained by SCNA detection tool is proportional to the subclonal frequency. For the samples with higher subclonal frequency, the “true” SCNA segments could be segmented into multiple segments, which causes the Fig. 3c and b presents the same stripe pattern.

For NGS based SCNA analysis, the read count ratio stripes could serve as a good proxy for bias correction, even if the break points are not correct, because the read count ratio of the “true” SCNA segment is preserved as the center of read count ratio stripe.

## Conclusion

Pre-SCNAClonal proposed in this paper is a robust GC bias correction and baseline selection tool for SCNAs based tumor subclonal inferring. Pre-SCNAClonal could correct the read count ratio’s GC bias and improve the performance of SCNA based subclonal inferring tools at all levels of tumor subclonal frequency even the subclonal frequency is very small. Furthermore, Pre-SCNAClonal also provides an user-friendly interface for visualizing and manually correcting the GC bias of read count ratio.

## Additional file

Additional file 1 Modeling and Correct the GC bias of tumor and normal WGS data for SCNA based tumor subclonal population inferring. (PDF 2570 kb)
