# Supplementary material for: Modeling and correct the GC bias of tumor and normal WGS data for SCNA based tumor subclonal population inferring
Source: BMC Bioinformatics. 2018 Apr 11;19(Suppl 5):112. doi: 10.1186/s12859-018-2099-0 (PMC5907144; doi:10.1186/s12859-018-2099-0)
Supplement: Supplementary file 1 — Modeling and Correct the GC bias of tumor and normal WGS data for SCNA based tumor subclonal population inferring. (PDF 2570 kb) [file 12859_2018_2099_MOESM1_ESM.pdf]

Modeling and Correct the GC bias of tumor and normal WGS data for SCNA based tumor subclonal population inferring

Chu Yanshuo[1], *

Supplementary Information

Contents

1 Supplementary figures **1**

2 Read count ratio bias simulation **3**
2.1 Method 3
2.2 The performance of Pre-SCNAClonal on simulation data 4

3 Baseline selection **6**
3.1 Hierarchy clustering based baseline selction model 6
3.2 Performance of baseline selection model of Pre-SCNAClonal and MixClone 6
3.3 Validation of baseline selected by Pre-SCNAClonal 6
3.3.1 The ploidy number 6
3.3.2 The BAF distribution on germline heterozygous SNP site 10

1 Supplementary figures

*corresponding author

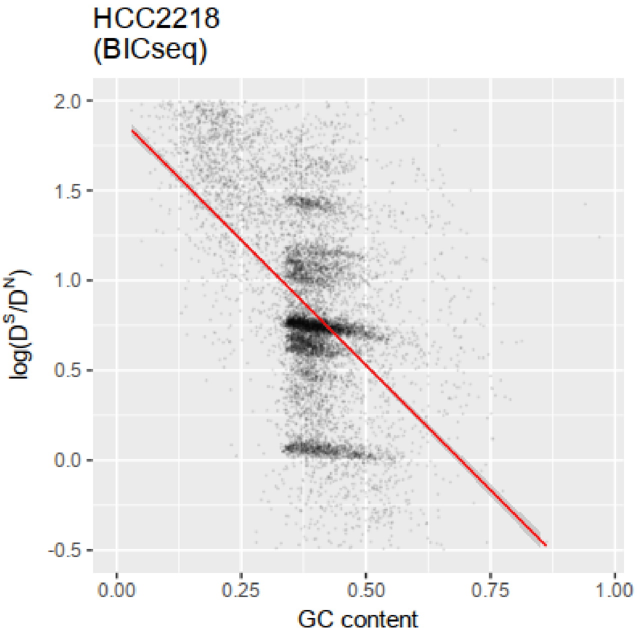

Figure 1: GC bias of read count ratio of paired tumor and normal sample of HCC2218. Red line denotes the linear regression line. SCNA segments are obtain by BIC-seq[5]. The WGS sequence alignment data (.bam files) of 'HCC2218' and its paired normal sample are publicly available on Illumina BaseSpace Sequence Hub website

2 Read count ratio bias simulation

2.1 Method

To further test Pre-SCNAClonal, we develop a package that could simulate data that presents stripes with log-linear bias pattern. It mainly contains two steps to generate simulation data, i) generate the data without any bias; ii) generate the biased data based on this data.

Given the total number of segments n, first we need to allocate n to stripes. We calculate all the possible average copy number $\bar{C}_k$ by

$$\bar{C}_k = \tau_k * C + (1 - \tau_k) * 2;\ C \in [1, cn_{\max}], k \in [1, sn], \tag{1}$$

here, $cn_{\max}$ is the maximum copy number pre-defined, sn is the subclonal number pre-defined, τ_k are pre-defined subclonal frequency for subclone k, C is the absolute copy number. Then we could get the number of stripes as the unique number of the value of all the possible average copy numbers (denoted as unique($\bar{\boldsymbol{C}}$)). Next we allocate n to unique($\bar{\boldsymbol{C}}$) stripes. The proportions of segments of all stripes $\boldsymbol{p}$ are sampled from a Dirichlet distribution,

$$\boldsymbol{p} \sim \mathrm{Dir}(\boldsymbol{\alpha}), \tag{2}$$

here, each value of the parameter vector $\boldsymbol{\alpha}$ equals 1, dimension of $\boldsymbol{p}$ and $\boldsymbol{\alpha}$ equals unique($\bar{\boldsymbol{C}}$).

Then we get the center of each strip $\mathrm{m}_{\boldsymbol{Y}_j}$ by,

$$\mathrm{m}_{\boldsymbol{Y}_j} = \log\left[\frac{\bar{C}_j}{2}\right];\ j \in [1, \mathrm{unique}(\bar{\boldsymbol{C}})], \tag{3}$$

here, we do not simulate the somatic deletions, for log operation causes the $\mathrm{m}_{\boldsymbol{Y}_j}$ of somatic deletions dispersed and approaching to negative infinite. Next the y coordinates of stripe j, $\boldsymbol{Y}_j$, could be sampled from normal distribution,

$$\boldsymbol{Y}_j \sim \mathrm{Normal}(\mathrm{m}_{\boldsymbol{Y}_j}, \sigma_j), \tag{4}$$

here, σ_j is obtained by

$$\sigma_j = \begin{cases} \frac{1}{2} * \eta * (\mathrm{m}_{\boldsymbol{Y}_{j+1}} - \mathrm{m}_{\boldsymbol{Y}_{j-1}}) & 1 < j < \mathrm{unique}(\bar{\boldsymbol{C}}) \\ \eta * (\mathrm{m}_{\boldsymbol{Y}_{j+1}} - \mathrm{m}_{\boldsymbol{Y}_j}) & j = 1 \\ \eta * (\mathrm{m}_{\boldsymbol{Y}_j} - \mathrm{m}_{\boldsymbol{Y}_{j-1}}) & j = \mathrm{unique}(\bar{\boldsymbol{C}}) \end{cases} \tag{5}$$

For stripe j, x coordinates are sample from a belta distribution, parameter a_j, b_j define its horizontal density distribution,

$$\zeta_j \sim \mathrm{Belta}(a_j, b_j), \tag{6}$$

here, ζ_j is the location between the max and min of $\boldsymbol{X}_j$, then x_{ji} is

$$x_{ji} = \zeta_{ji} * [\max(\boldsymbol{X}_j) - \min(\boldsymbol{X}_j)] + \min(\boldsymbol{X}_j). \tag{7}$$

Generally, we set

$$\min(\boldsymbol{X}_j) = 0.35, \tag{8}$$

and

$$\max(\boldsymbol{X}_j) = \begin{cases} 0.85 & if\ \bar{C}_j = 2, \\ \left(\mathrm{m}_{\boldsymbol{Y}_j} - c_{\mathrm{up}}\right) * \frac{1}{m_{\mathrm{up}}} & if\ \bar{C}_j > 2, \\ \left(\mathrm{m}_{\boldsymbol{Y}_j} - c_{\mathrm{down}}\right) * \frac{1}{m_{\mathrm{down}}} & if\ \bar{C}_j < 2. \end{cases} \tag{9}$$

Next, the number of the segments in stripe j with higher GC content is less than the segments with lower GC content, we set $1 < a_j < b_j$ in Equation 6 and shrink y_{ji} by

$$y_{ji} = \left[s_j * \frac{\max(\boldsymbol{X}_j) - x_{ji}}{\max(\boldsymbol{X}_j) - \min(\boldsymbol{X}_j)} + (1 - s_j)\right](y_{ji} - \mathrm{m}_{\boldsymbol{Y}_j}) + \mathrm{m}_{\boldsymbol{Y}_j}, \tag{10}$$

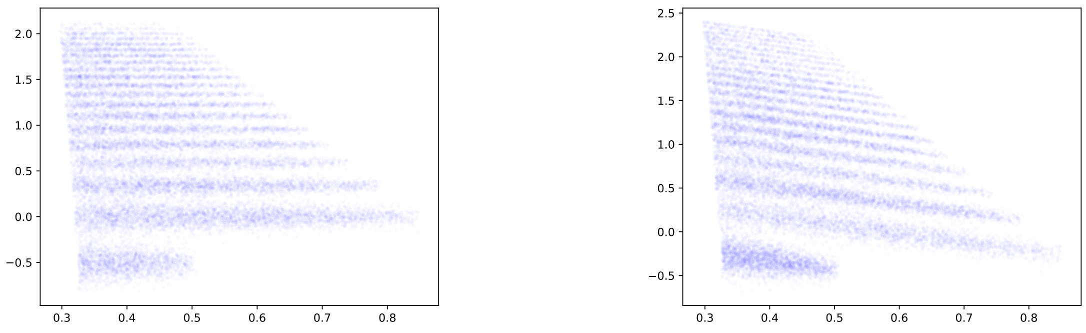

(a) Simulation data without bias.

(b) Simulation data with bias.

Figure 2: Simulation data generated by simulation package. a) The original data without bias. it is generated with maximum copy number assigned as 20, subclonal number assigned as 1, purity assgned as 0.8. b) The simulation data with GC bias, which is generate from a) with horizontal bias slope set as -1, vertical bias slope assigned as -90.

here $s_j \in (0, 1)$ is the shrink factor pre-defined for stripe j.

Final step, assign bias to the original data. The vertical bias is generated by

$$x_{ji} = x_{ji} + (\mathrm{m}_{\boldsymbol{Y}_j} - c_v)/m_v, \tag{11}$$

given the horizontal bias slope m_v and interception c_v. And the horizontal bias is generated by

$$y_{ji} = y_{ji} - (m_h * x_{ji} + c_h) + \mathrm{median}(\boldsymbol{Y}), \tag{12}$$

given the vertical bias slope m_h,and interception c_h.

2.2 The performance of Pre-SCNAClonal on simulation data

We simulate the $\log(D^S/D^N)$ data with maximum copy number 20, subclonal number 1, purity 0.8, as shown in Figure 2a. Then we apply GC bias to this simulated data with vertical GC bias slope -90, horizontal GC bias from -20 to 20. For each combination of vertical and horizontal GC bias, we apply MCMC correction model to correct the horizontal GC bias 10 times. As shown in Figure 3, the boxplot of GC correction result of MCMC model shows that most of horizontal GC bias slope predicted by MCMC model is precisely the horizontal GC bias pre-set, while the linear regression model could not correctly predicted the horizontal GC bias slope.

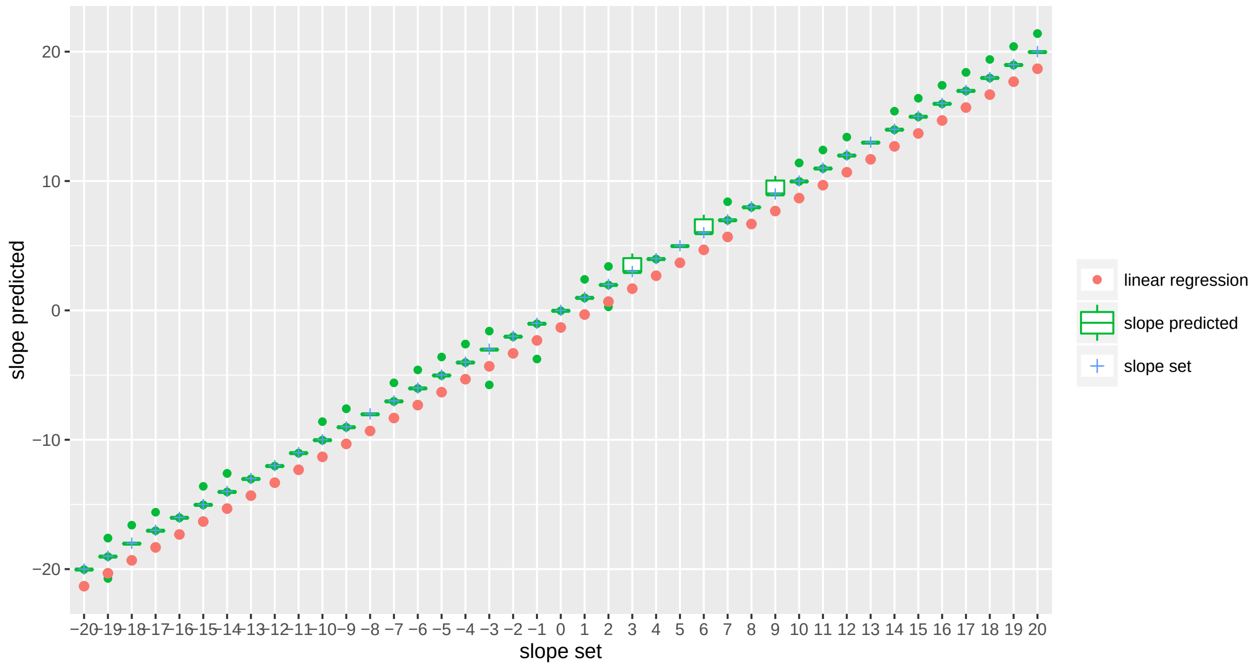

Figure 3: Result of MCMC model and linear regression model on simulation data.

3 Baseline selection

3.1 Hierarchy clustering based baseline selction model

SCNAs based subclonal reconstruction tools, such as MixClone[3] and THetA[4], use the read coverage and B-allele frequency (BAF) together to estimate the absolute copy number and population frequency. Since there exists difference between the WGS data of tumor-normal paired samples (such as batch effects), the $\log(D^S/D^N)$ of baseline segments not always locate at 0. MixClone[3] tries to detect the baseline segments through BAF information while THetA[4] leaves this problem to user. MixClone[3] assumes that the average value of segments which are not Lost of Heterzyosity (LOH) segments is the baseline location. This assumption is not correct for the case that all the segments with positive number of the maternal copy and paternal copy, are not LOH segments.

Here we propose the features of baseline segments,

- the segment does not lose heterzyosity;
- the number of maternal copy and the number of paternal copy of the segment are the same;
- for all the segments meet above two conditions, the segments with the minimum $\log(D^S/D^N)$ are baseline segments.

The top two conditions limit the segments to even copy number strips, then the last condition pick out segments with the minimum even copy number 2 as the baseline segments. Pre-SCNAClonal uses the same method in MixClone[3] to get the LOH status of every segment. Next, for each segment, Pre-SCNAClonal select the segments that contain loci of heterzyogous SNPs with 50% B-allele frequency in sequencing data of tumor sample from the LOH segments, denoted as APM (Average Paternal Maternal) segments. Then Pre-SCNAClonal use hierarchy clustering model to group APM segments according to their $\log(D^S/D^N)$ value. Finally, Pre-SCNAClonal select the segment group with the minimum $\log(D^S/D^N)$ value as baseline segments.

Generally, there are two typical methods to obtain the BAF sites of each segment, i) find out all the heterzyogous sites from aligned WGS read set of normal sample (i.e. MixClone[3]); ii) use the pre-known heterzyogous SNP sites as input, filter out the heterzyogous sites from aligned WGS read set of normal sample (i.e. THetA[4]); The number of the heterzyogou SNP sites detected by the second method is equal or less than the number detected by the first method. Pre-SCNAClonal imposes another limitation, the density threshold of heterzyogou SNP in each segment, to filer out highly reliable baseline segments.

3.2 Performance of baseline selection model of Pre-SCNAClonal and MixClone

MixClone[3] obtains baseline by removing outliers of the segments that do not lose heterzygosity. For sequencing data, it is difficult to distinguish LOH from sequencing deviation. As shown in Figure 4, segments that do not lose heterzygosity are randomly distributed everywhere. Baseline selection method of MixClone almost picks out all the segments as baseline while the tumor purity is low. In comparison with baseline obtained by MixClone, as shown in Figure 5, baseline obtained by Pre-SCNAClonal is lower and more consistent than the baseline obtained by MixClone.

3.3 Validation of baseline selected by Pre-SCNAClonal

3.3.1 The ploidy number

According to the multiple studies as shown in Table 1, tumor sample HCC1954 is tetraploidy. In this subsection, we calculate the ploidy number based on baseline position to validate baseline selection model of Pre-SCNAClonal.

Figure 6 shows the distribution of $\log(D_i^S/D_i^N)$ of the paired tumor-normal sample of HCC1954 (coverage 58x, purity 99%). We manually pick out the discernible stripes, marked with 'a', 'b', 'c', 'd', 'e', then we mark all the segments lower than 'a' with 'a" and mark all the segments higher than 'e' with 'f'. Then we manually get the upper boundary and lower boundary of each stripes listed in Table 2. According to Equation **??** in Section **??**, the distance between stripes with high copy number approaches 0. For the indiscernible area 'f',

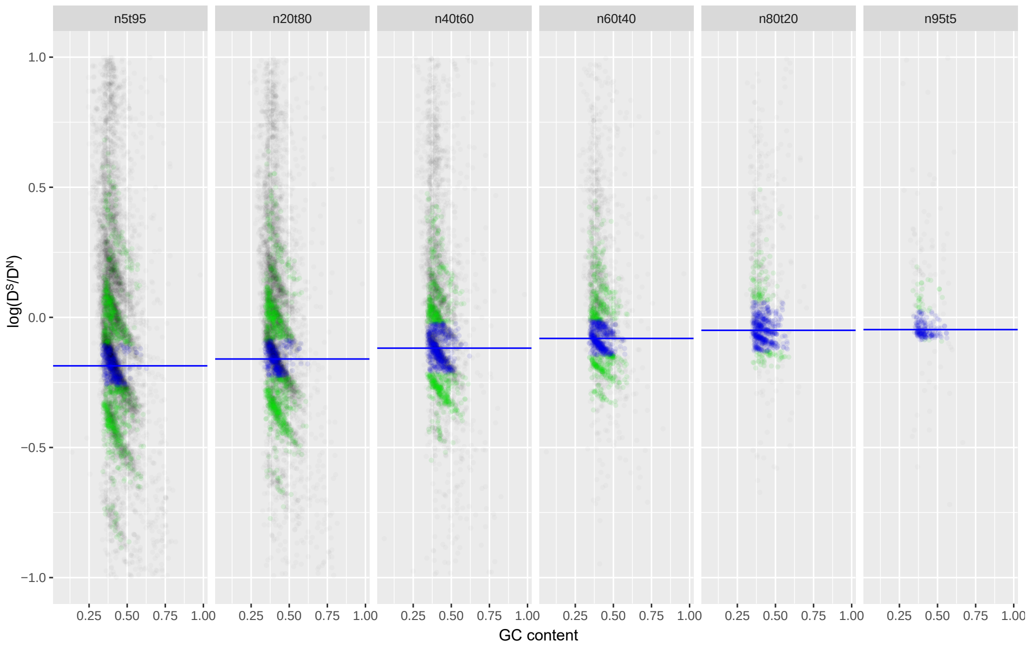

Figure 4: Distribution of uncorrected log D^S/D^N and baseline segments selected by MixClone. The green and blue points together are the segments with no LOH, the blue points are the baseline segments selected by removing the segments with outlier value of D^S/D^N from segments with no LOH, here the all the points are plotted by ggplot2 R package with opacity parameter $\alpha = 0.05$. The blue line in each sub-figure is the average value of D_j^S/D_j^N of baseline segments.

Table 1: Ploidy of tumor sample HCC1954 predicted by different tools

Tools	COSMIC[2]	ABSOLUTE[1]
Ploidy	4.2	4.5

Table 2: Boundary of stripes in Figure 6

Stripe	a'	a	b	c	d	e	f
Upper boundary (log $\left(\frac{D_i^S}{D_i^N}\right)$)	-0.7	-0.4	-0.1	0.15	0.345	0.5	∞
Lower boundary (log $\left(\frac{D_i^S}{D_i^N}\right)$)	$-\infty$	-0.7	-0.4	-0.1	0.15	0.345	0.5
Absolute copy number if'c' is baseline	0	0	1	2	3	4	5
Absolute copy number if'b' is baseline	0	1	2	3	4	5	6
Absolute copy number if'a' is baseline	1 or 0	2	3	4	5	6	7

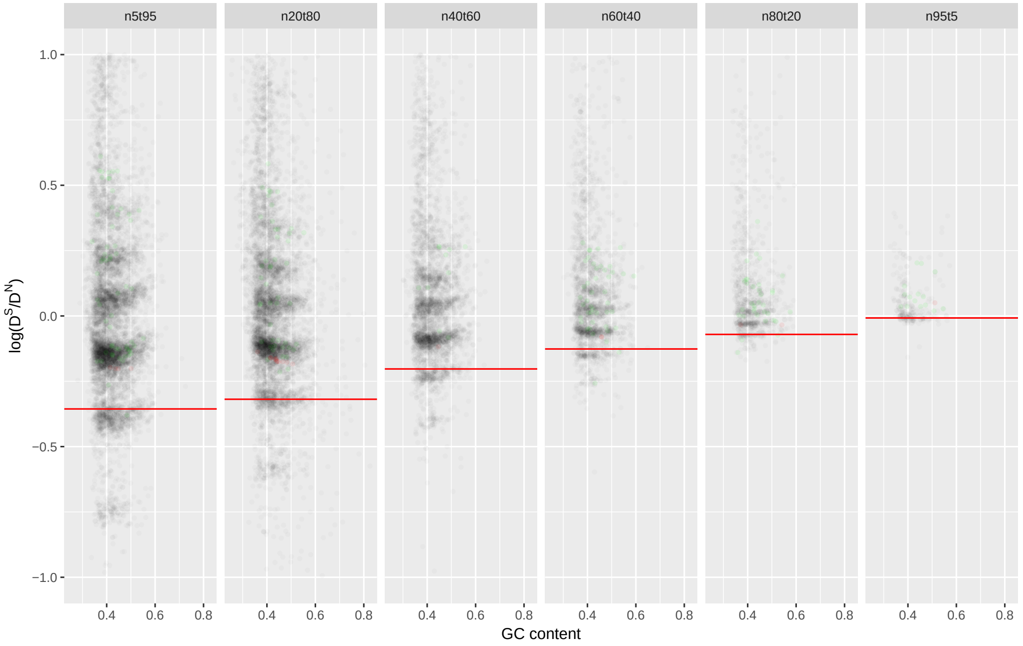

Figure 5: Distribution of corrected $\log D^S/D^N$ and baseline segments selected by Pre-SCNAClonal. The green and blue points together are the segments with no LOH and B allele frequencies around 0.5 (APM segments), the red points are the baseline segments selected by method proposed in this study which is clustering the value $\log D^S/D^N$ from segments with no LOH and B allele frequencies around 0.5, then selecting the segments with lowest average $\log D^S/D^N$ as baseline. All the points in this figure are plotted by ggplot2 R package with opacity parameter $\alpha = 0.05$. The red line in each sub-figure is the average value of $\log D^S/D^N$ of baseline segments.

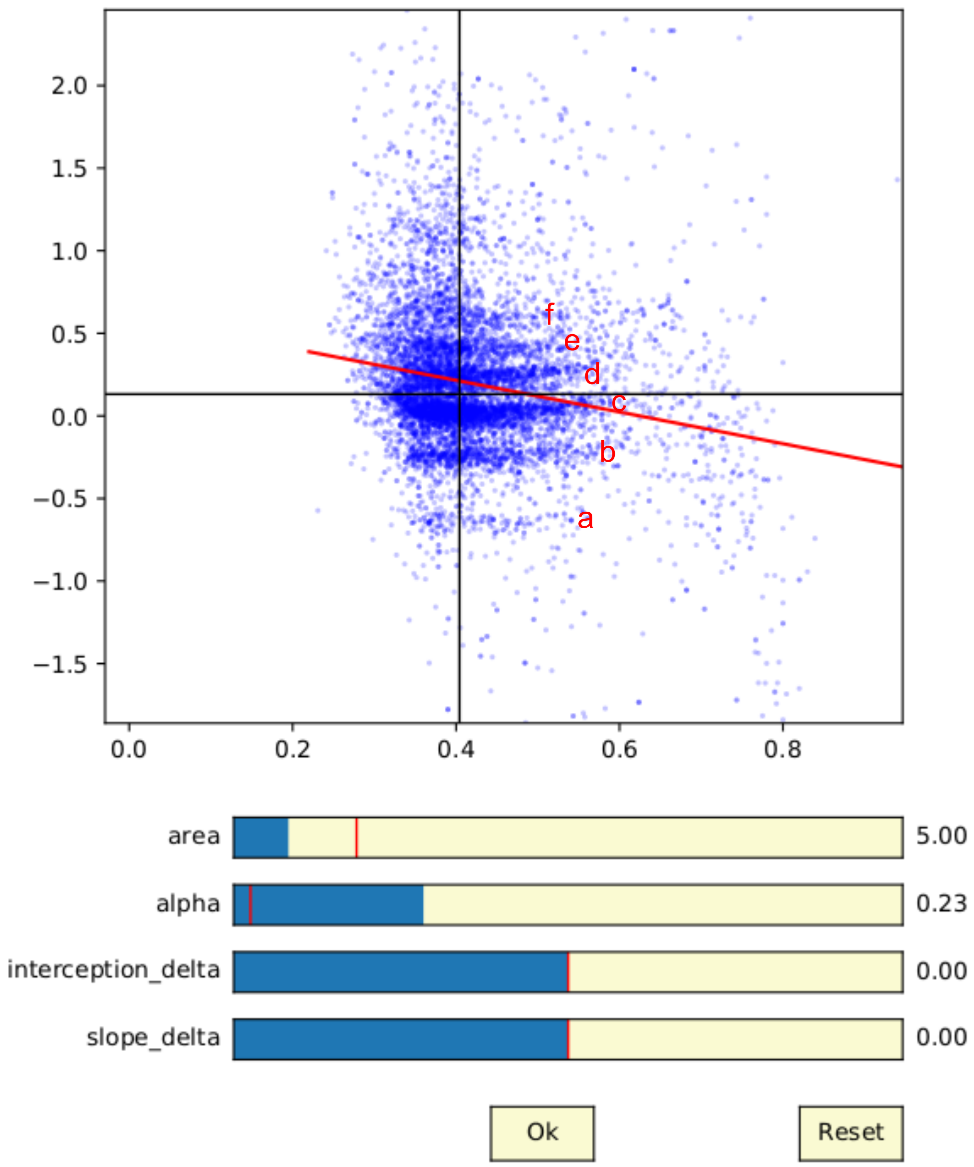

Figure 6: The distribution of $\log(D_i^S/D_i^N)$ of tumor sample HCC1954 (coverage 58x, purity 99%).

we use Equation ?? to estimate the absolute copy number of each segment in it. We use stripe ‘e’ to correct the segments location, to make all the absolute copy numbers of segments in ‘f’ larger than the absolute copy number of stripe ‘e’. Then we calculate the plodiy number ξ by

$$\xi = \frac{\sum_{i}^{n}(cn_i * l_i)}{\sum_{i}^{n} l_i}. \tag{13}$$

Here, n denotes the total segment number, cn_i denotes the absolute copy number of segment i and l_i denotes the segment length of segment i.

Table 3: Ploidy number estimated based on different baseline locations

Baseline location	Ploidy number ξ
Stripe ‘c’	2.721
Stripe ‘b’	3.876
Stripe ‘a’(cn of ‘a” is 0)	5.144
Stripe ‘a’(cn of ‘a” is 1)	5.142

As shown in Table 3, the ploidy number is 3.876 while stripe ‘b’ is baseline, which is closer to the results obtained by the previous studies listed in Table 1 than the ploidy number based on the other two baselines.

3.3.2 The BAF distribution on germline heterozygous SNP site

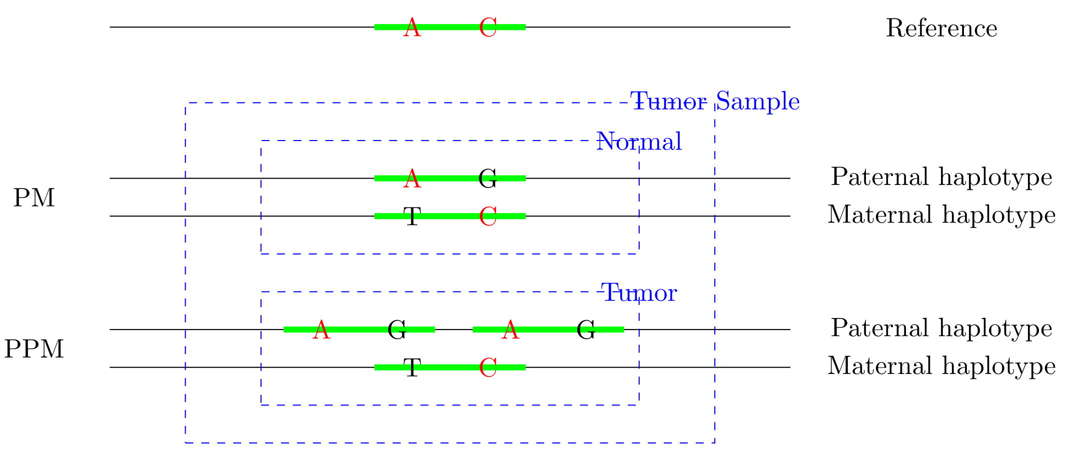

Figure 7: B allele on the germline heterozygous SNP site. The definition of B allele is the allele that is different from reference allele. In this figure, “T” and “G” are B alleles. In this figure, tumor sample contains one tumor cell and one normal cell (dashed blue line). There is a SCNA in the tumor cell, absolute copy number is 3 (The green lines denote the SCNA segments on the DNA chains).

Let μ_i denote the BAF of SCNA segment i of tumor genome on germline heterozygous SNP site, and let C_i, G_i respectively denote the absolute copy number and genotype of SCNA segment i. As shown in Figure 7, the B allele (non-reference allele) could be either maternal or paternal allele, thus the BAF of SCNA segments of tumor genome presents symmetrical pattern in $[0, 1]$ (as shown in Table 4). Let ξ_i denote the BAF of the tumor sample, ϕ_i denote the subclonal population frequency, then,

$$\xi_i = \frac{\phi_i * C_i * \mu_i + (1 - \phi_i) * 2 * \frac{1}{2}}{\phi_i * C_i + (1 - \phi_i) * 2}. \tag{14}$$

Table 4: B allele frequency distribution of tumor genome on germline heterozygous SNP site.

C_i	G_i	μ_i
0	$\oslash$	–
1	P	1
1	M	1
2	PP	0 or 1
2	PM	$\frac{1}{2}$
2	MM	1 or 0
3	PPP	0 or 1
3	PPM	$\frac{1}{3}$ or $\frac{2}{3}$
3	PMM	$\frac{2}{3}$ or $\frac{1}{3}$
3	MMM	1 or 0
...	...	...

In Equation 14, '2' and '$\frac{1}{2}$' are the copy number and heterozygous BAF of normal sample. Then, ξ_i is symmetrical in $[0, 1]$, because μ_i is symmetrical in $[0, 1]$.

Since 'HCC1954.mix.n20t80' contains only one subclone, and the average copy number of each segment in the same stripe is the same, then each stripe contains only one absolute copy number (according to Equation **??**). So the number of paternal allele and the number of maternal allele at the heterozygous SNP sites in the segments with the odd absolute copy number are not equal. As shown in Figure 8, the BAF distritions in stripe 'b'–'f' of uncorrected segments present only one peak at 0.5, while the BAF distritions in stripe 'e', 'f' of corrected segments present 2 or 3 peaks. It proves that the GC bias correction method of Pre-SCNAClonal is effective.

The BAF distribution of all the segments in stripe 'a' corrected by Pre-SCNAClonal presents two peaks at around 0.2 and 0.8, according to Equation 1, the absolute copy number should be 1, therefore stripe 'b' is baseline.

Only the allele types with the same genotype could cause the tumor BAF distribution on paired normal heterozygous allele loci presents symmetrical peaks. For example, BAF of genotype "PPMMM" could be 2/5 or 3/5, because the reference allele could be either paternal or maternal. Figure 8 also reveals that SCNAs in the same stripe possess the same absolute copy number.

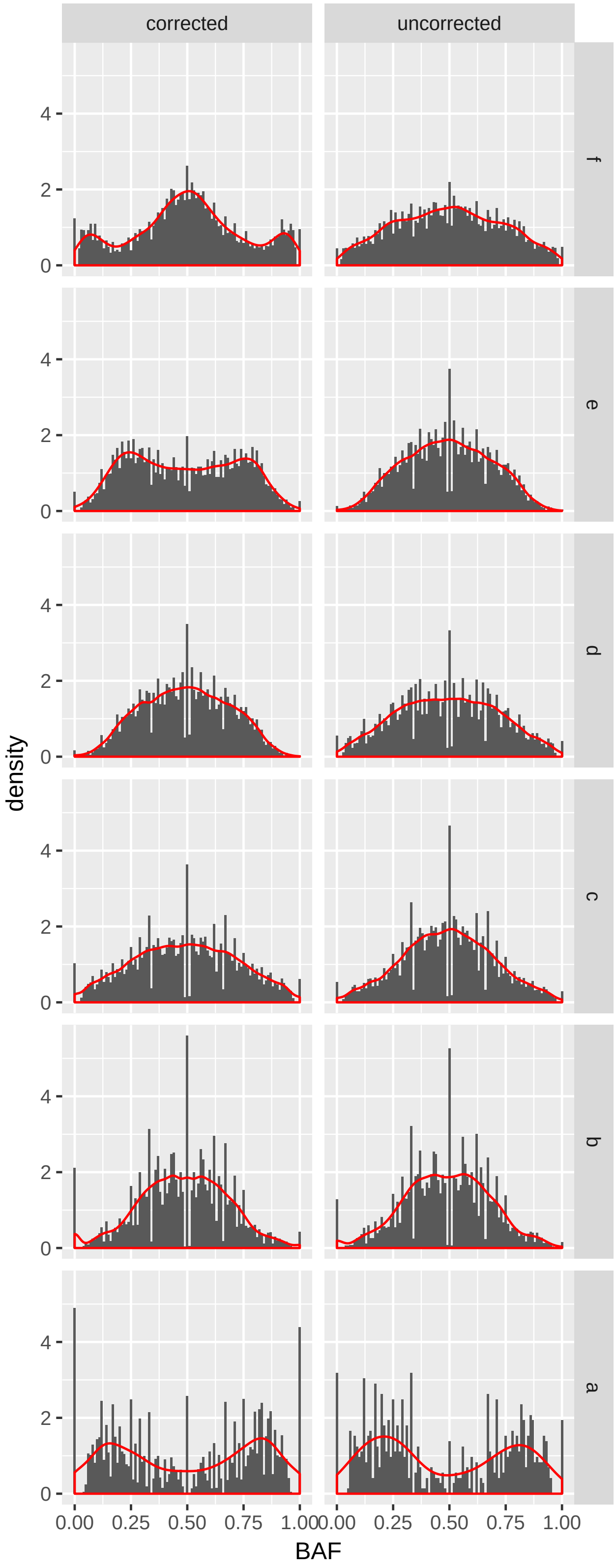

Figure 8: BAF distribution in stripe 'a'–'f' of 'HCC1954.mix.n20t80'. Figures on the left side are BAF distributions of all the segments in each stripe corrected by Pre-SCNAClonal while figures on the right side are BAF distributions of all the segments at the same location that without GC correction.

References

[1] Carter, S. L., Cibulskis, K., Helman, E., McKenna, A., Shen, H., Zack, T., Laird, P. W., Onofrio, R. C., Winckler, W., Weir, B. A., *et al.* (2012). Absolute quantification of somatic dna alterations in human cancer. *Nature biotechnology*, **30**(5), 413–421.

[2] Forbes, S. A., Beare, D., Gunasekaran, P., Leung, K., Bindal, N., Boutselakis, H., Ding, M., Bamford, S., Cole, C., Ward, S., *et al.* (2015). Cosmic: exploring the world's knowledge of somatic mutations in human cancer. *Nucleic acids research*, **43**(D1), D805–D811.

[3] Li, Y. and Xie, X. (2015). Mixclone: a mixture model for inferring tumor subclonal populations. *BMC genomics*, **16**(Suppl 2), S1.

[4] Oesper, L., Mahmoody, A., and Raphael, B. J. (2013). Theta: inferring intra-tumor heterogeneity from high-throughput dna sequencing data. *Genome biology*, **14**(7), 1.

[5] Xi, R., Luquette, J., Hadjipanayis, A., Kim, T.-M., and Park, P. J. (2010). Bic-seq: a fast algorithm for detection of copy number alterations based on high-throughput sequencing data. *Genome biology*, **11**(1), 1.
